# Mapping and Modeling of Discussions Related to Gastrointestinal Discomfort in French-Speaking Online Forums: Results of a 15-Year Retrospective Infodemiology Study

**DOI:** 10.2196/17247

**Published:** 2020-11-03

**Authors:** Florent Schäfer, Carole Faviez, Paméla Voillot, Pierre Foulquié, Matthieu Najm, Jean-François Jeanne, Guy Fagherazzi, Stéphane Schück, Boris Le Nevé

**Affiliations:** 1 Innovation Science and Nutrition Danone Nutricia Research Palaiseau France; 2 Kap Code Paris France; 3 Deep Digital Phenotyping Research Unit Department of Population Health Luxembourg Institute of Health Strassen Luxembourg; 4 Center of Research in Epidemiology and Population Health UMR 1018 Inserm, Institut Gustave Roussy Paris-Sud Paris-Saclay University Villejuif France

**Keywords:** gastrointestinal discomfort, disorders of gut-brain interactions, social media, infodemiology, topic modeling

## Abstract

**Background:**

Gastrointestinal (GI) discomfort is prevalent and known to be associated with impaired quality of life. Real-world information on factors of GI discomfort and solutions used by people is, however, limited. Social media, including online forums, have been considered a new source of information to examine the health of populations in real-life settings.

**Objective:**

The aims of this retrospective infodemiology study are to identify discussion topics, characterize users, and identify perceived determinants of GI discomfort in web-based messages posted by users of French social media.

**Methods:**

Messages related to GI discomfort posted between January 2003 and August 2018 were extracted from 14 French-speaking general and specialized publicly available online forums. Extracted messages were cleaned and deidentified. Relevant medical concepts were determined on the basis of the Medical Dictionary for Regulatory Activities and vernacular terms. The identification of discussion topics was carried out by using a correlated topic model on the basis of the latent Dirichlet allocation. A nonsupervised clustering algorithm was applied to cluster forum users according to the reported symptoms of GI discomfort, discussion topics, and activity on online forums. Users’ age and gender were determined by linear regression and application of a support vector machine, respectively, to characterize the identified clusters according to demographic parameters. Perceived factors of GI discomfort were classified by a combined method on the basis of syntactic analysis to identify messages with causality terms and a second topic modeling in a relevant segment of phrases.

**Results:**

A total of 198,866 messages associated with GI discomfort were included in the analysis corpus after extraction and cleaning. These messages were posted by 36,989 separate web users, most of them being women younger than 40 years. Everyday life, diet, digestion, abdominal pain, impact on the quality of life, and tips to manage stress were among the most discussed topics. Segmentation of users identified 5 clusters corresponding to chronic and acute GI concerns. Diet topic was associated with each cluster, and stress was strongly associated with abdominal pain. Psychological factors, food, and allergens were perceived as the main causes of GI discomfort by web users.

**Conclusions:**

GI discomfort is actively discussed by web users. This study reveals a complex relationship between food, stress, and GI discomfort. Our approach has shown that identifying web-based discussion topics associated with GI discomfort and its perceived factors is feasible and can serve as a complementary source of real-world evidence for caregivers.

## Introduction

### Background

#### Gastrointestinal Discomfort: Prevalence, Impact on Quality of Life, and Management

Gastrointestinal (GI) discomfort (eg, bloating, abdominal pain, constipation) is very common in the general population, with a known impact on well-being [1]. Chronic and severe symptoms of GI discomfort are associated with a significant decrease in quality of life [2]. Irritable bowel syndrome (IBS) is the most studied condition among disorders of gut-brain interactions (DGBIs) [3], with a highly heterogeneous prevalence ranging from 1.1% in France and Iran to 35.5% in Mexico [4,5]. Associated socioeconomic costs are significant because of the important use of health care resources and work absenteeism [6-8]. However, the etiology of DGBIs remains to be poorly understood. Among the pathophysiological mechanisms associated with IBS, GI sensory-motor alterations [9,10], signs of discrete immune dysfunction [11], and increased intestinal permeability [12] are considered important. The possible involvement of gut microbiota in the pathogenesis of GI diseases and the occurrence of GI symptoms has also been explored, as the severity of IBS symptoms is associated with specific intestinal microbiota profiles [13].

Psychological comorbidities are commonly associated with GI symptoms, and the prevalence of anxiety and depression among people with IBS is estimated to be at least two to three times the rate in the general population [14,15]. Concerning women with abdominal pain, cramping, and discomfort, a recent web-based cross-sectional survey study [16] showed that 96 % of women reported that daily activities were disrupted at least sometimes by abdominal pain, cramping, and discomfort and 44 % of women reported that daily activities were disrupted at least often. Other aspects of quality of life, such as quality of work, eating habits, and social activities were also affected in most women [16].

DGBIs are the source of important health care consumption (consultations, complementary examinations and hospitalizations), although difficult to quantify [17], given their chronic nature and the absence of specific diagnostic tests in the case of IBS [18]. In France, a cross-sectional study estimated the average annual direct cost to be €756 (US $888) for one patient and more than 3 days of sick leave per year [17]. Another study conducted in the United Kingdom [19] estimated the total cost of DGBIs in infants to be at least £72.3 (US $93.7) million per year in 2014 to 2015, of which £49.1 (US $63.6) million was the National Health Service expenditure on prescriptions, community care, and hospital treatment.

The role of diet in the pathogenesis of IBS has already been highlighted [20], and food is perceived as a factor of GI discomfort even in the absence of diagnosed allergy or malabsorption [21]. The management of DGBIs especially relies on lifestyle, including physical activity and dietary measures. Available guidelines [22] recommend regular meal patterns, avoidance of large meals, and reduced intake of fat, alcohol, spicy foods, insoluble fibers, caffeine, and gas-producing foods such as beans, cabbage, and onions. Eating meals in a quiet place (for about at least 20 min, without working) with sufficient chewing and hydration (1.5 to 2 liters per day) is also recommended [22]. Dietary interventions (probiotics, prebiotics, and synbiotics) and restriction diets (eg, low-fermentable oligosaccharides, disaccharides, monosaccharides, and polyols, also known as low-FODMAP diet) have also been explored as potential therapeutic solutions in IBS [23]. Available pharmacological treatments targeting either the GI tract or the brain have also shown some therapeutic value and include antidepressants, prokinetic agents, and painkillers [24].

#### Social Media as a Real-World Health Data Source

The penetration of social media into modern society has become a global cultural phenomenon. Patients use peer-to-peer virtual communities and social media to share their experiences regarding their treatments and diseases. The use of social media allows large groups of people to create and share information, opinions, and experiences about health conditions and medications through web-based discussion [25]. Social media can therefore be considered as a new data source to assess population health and quality of life, understand adherence to treatments, or identify adverse drug reactions. Patients highlighted the benefit of web-based interactions with other patients. For example, sharing information through social networks enabled patients to better communicate with health care providers. Patients often use social media to discuss drug side effects, quality of life and adherence to therapies. To analyze such data, which can be voluminous, appropriate tools are needed. Text mining techniques allow the classification and summarization of text data such as messages [26]. This set of techniques has been used to extract information from electronic health records [27,28]. They have also been used for various use cases with social media data. Some authors [29] studied the messages from patients with breast cancer treated with aromatase inhibitors. In France, some studies have been published on the misuse and pharmacovigilance signals of methylphenidate [30,31], the incorrect use of neuroleptics regarding anxiety [32], the safety profile of Levothyrox and the dynamics of its reporting on social media during the summer of 2017 [33]. In multiple therapeutic areas, including diabetes and obesity, social media has been considered a real-world health evidence data source [34]. Although web-based discussions are unstructured as compared with conventional clinical data, their volume (hundreds of thousands of users) is very important when compared with clinical sets, and this information can therefore be considered as a complementary source of health data in observational research.

### Objectives

This study was designed to explore perceived GI discomfort and better understand its determinants, on the basis of a retrospective assessment of web-based social media posts, which we considered as a real-life source of health information. In this study, we aimed to answer 3 main research questions: (1) Can we identify topics discussed by web users reporting symptoms of GI discomfort? (2) Can we categorize these users on the basis of the reported symptoms of GI discomfort and level of activity on social media while considering their age and gender? (3) Can we identify the perceived factors causing GI discomfort as reported by web users?

## Methods

### Data Sources and Data Extraction

**Figure 1:**
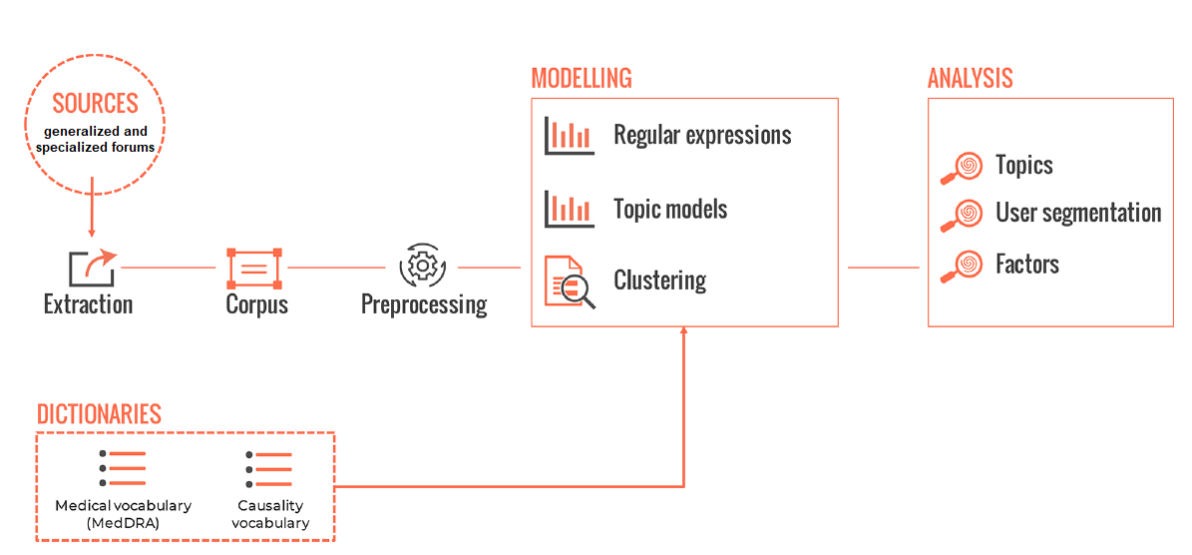
Study framework.

Messages were retrieved (Figure 1) from general and specialized French medical web forums. Only messages from publicly available sources were extracted [35]. Messages published between January 2003 and August 2018 containing keywords related to GI discomfort were retrieved along with messages from 3 GI discomfort–related subforums of *Doctissimo* [36]: (1) *constipation, other transit disorders*; (2) *digestion, heartburn, gastroesophageal reflux disease (GERD);* and (3) *abdominal pain, stomachache and ulcers*. Messages were automatically extracted using the published *Detec’t webcrawler* [37,38] developed by Kap Code. A web crawler is an engine that browses through hyperlinks and stores them for future download of associated web pages (identified by the visited hyperlinks) [39]. Scraping of messages was performed according to the HTML structure of each forum. All discussions containing at least one of the keywords or one of their synonyms were automatically retrieved with all the associated metadata, deidentified and cleaned (signature and quote withdrawal) before being stored in a study-specific database. A description of the extracted corpus is presented in Multimedia Appendix 1. A complete list of the forums that were crawled is presented in Multimedia Appendix 2, and a list of the keywords used for message retrieval is detailed in Multimedia Appendix 3.

The corpus is presented in number of messages, one message being one statistical unit. As the total number of extracted messages could not be estimated in advance, no a priori assumption was made about the topics being discussed by web users, the clusters to be segmented, or the perceived factors to be identified, and no sample size was calculated for this observational study.

### Data Exclusion

**Figure 2:**
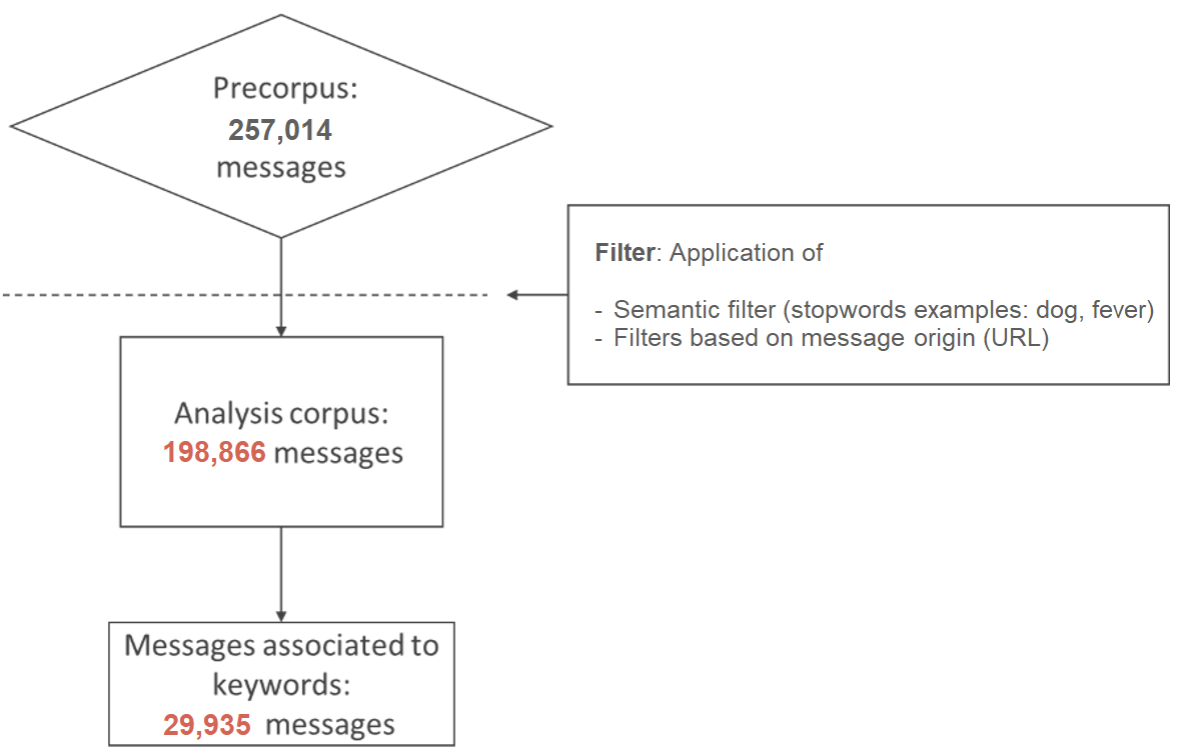
Flowchart presenting message extraction.

The analysis corpus consisted of the corpus cleaned after the removal of messages containing predetermined keywords written in a language other than French, messages containing at least one of the study-specific exclusion words (such as animal-related vocabulary or GI symptoms being used out of context), messages coming from specific URLs and duplicates, as presented in Figure 2.

### Statistical Analyses

#### Discussion Themes and Topics

A topic model was applied to identify the themes addressed in the messages. Topic models consist of text mining approaches that aim to automatically identify the abstract themes addressed in a collection of documents. The simplest and most current form of topic models is latent Dirichlet allocation (LDA) [40,41]. It is based on the hypothesis that each document in the corpus corresponds to a distribution of several topics, these distributions being Dirichlet prior. The modeled topics are probability distributions over the tokens (words or a sequence of several adjacent words) found in the corpus. There is no prior assumption made about the nature of topics present in the corpus under study. These models have already been used to analyze health-related topics within tweets [40,41] or online forums [29,42,43].

For this study, the correlated topic model was used [44,45]. In addition to being based on LDA [44], it considers the existing relations between discussed topics as an additional parameter. The estimated correlation between 2 topics indicates the extent to which these 2 topics emerged simultaneously in posts.

The modeling of the studied corpus went through different steps so that the topic model could be applied [30]. The model was estimated using a variational expectation maximization algorithm [44,45], which approximates the posterior distribution of topics on the corpus by finding the best combination of variational parameters. Topics being probability distributions over tokens of the study corpus, they can be characterized by the highest per-topic probability tokens. Weighting these probabilities through term frequency-inverse document frequency (TF-IDF) allows the allocation of higher importance to topic-specific tokens [45]. In this case, the per-topic probability of a token was weighted by the inverse of the probabilities of this token in other topics. For each topic, tokens were ranked from highest to lowest weighted TF-IDF value of their probability in this topic [45]. For each topic, the first 15 tokens obtained through this ranking were considered the most associated tokens. These were defined as the set of characteristic tokens and used to label each topic. This label should be a synthesis of the characteristic tokens expressed. Correlations between the different topics were measured. Topics were considered associated when correlations were higher than 0.2 in absolute value. This threshold has been set empirically to allow a post to be associated with 5 topics at most. Discussion topics were merged in *groups* of topics on the basis of the values of correlations, and some focus was on categories of interest by applying a new topic model to the associated messages. The analysis was performed using the Structural Topic Model package [46] with R environment version 3.5.2.

#### User Segmentation

A nonsupervised clustering algorithm (agglomerative hierarchical clustering) was applied on the data to categorize users according to their activity profile, using 36 different features, from 3 categories: symptoms of GI discomfort, identified topics, and website activity. These categories and features are presented in Multimedia Appendix 4.

A specific list of symptoms related to GI discomfort was established on the basis of the Medical Dictionary for Regulatory Activities (MedDRA) terms and colloquial language [37]. For this, a review of the medical dictionary, MedDRA version 15.0, was performed to identify all the terms that may be associated with GI discomfort. Subsequently, these terms were manually grouped by anatomical region or pathophysiological mechanism (esophageal disorders, gastric disorders, GI disorders, pain, appetite disorder, etc). A list of these regions and mechanisms used to group these messages is presented in Multimedia Appendix 5. Fifteen different categories of symptoms were established. A manual enrichment of these groups was made using colloquial language. Automatic screening of messages allowed the identification of specific GI symptoms expressed by web users.

Website activity of users was measured through different features such as the number of messages, the number of discussions, the dates of the first and last post, the forum name, or the mean posting span. Proportions of posts associated with categories of topics identified for the first objective were considered as the last type of features to describe web users. Created clusters of users were described via identity cards presenting the features that allowed to single them out with their age and gender distribution.

Web users’ gender was determined through the identification of regular expressions (gendered past participles, adjectives, and names) in messages and the application of a support vector machine on the basis of message content. This method achieves 88% accuracy and is the subject of a pending publication. Web user age categories were identified on the basis of the use of regular expressions of the author’s age in the messages, such as *J’ai 45 ans* (*I am 45 years old*). Each pseudo was associated with one gender (male, female, or unknown) and one age category (20 years or younger, 21-30 years, 31-40 years, 41-50 years, 51-60 years, 61 years or older, and unknown). The generated identification cards (features, age, and gender) were used to characterize each cluster and evaluate whether these characteristics were homogenous between clusters.

#### Factors of GI Discomfort

Factors perceived as responsible for the reported symptoms of GI discomfort were identified using a mixed automated analysis method combining syntactic analysis and topic model. The syntactic analysis was designed to identify (1) the messages containing extracted keywords and a causality term and (2) the sentences and phrases where the causality terms are present. To identify causality, a specific dictionary made of terms associated with causality was created. Causality terms consisted of terms or groups of terms expressing causality in French. These terms could be verbs conjugated at different times and pronouns (eg, *me donne* [makes me], *lui provoquait* [caused/triggered], *entrainent* [lead to/cause], etc), prepositions (eg, *à cause de* [because of/due to], etc), and conjunctions (eg, *dès lors* [since/consequently], *du fait de* [given that], etc).

**Figure 3:**
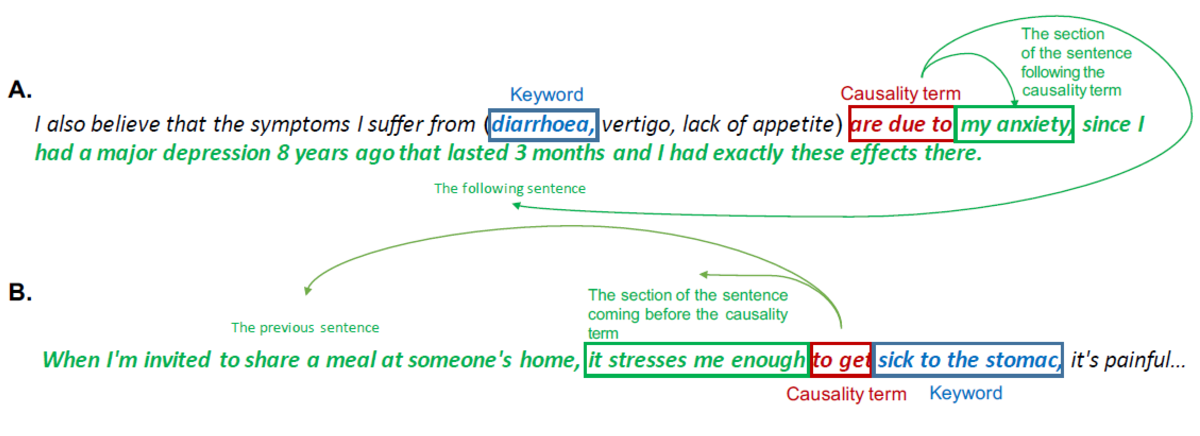
Example of messages. (A) Causality term associated with right section. (B) Causality term associated with left section. The sections in which the topic model is applied are indicated in green.

Depending on the causality terms, the position of the segment of phrases where factors could be identified was located either before or after the term, as presented in Figure 3. A topic model was then applied to the sections of messages depending on the direction associated with each causality term. These terms are presented in Multimedia Appendix 6 along with the associated direction of the segment of phrases to be analyzed. The number of topics was set to 30 to maximize the number of topics associated with factors that could arise. These topics were reviewed manually, and the topics of interest related to factors were identified.

## Results

### Data Set Description

After cleaning and formatting, the obtained corpus contained 198,866 messages. A total of 36,989 different web users were associated with this corpus. A total of 29,935 messages (corresponding to 16,746 different web users) contained at least one of the extraction keywords, and 181,365 messages came from the *Doctissimo* subforums (Multimedia Appendix 2).

The most frequent keyword was *abdominal pain,* as presented in Table 1. The most frequently mentioned keywords were lay vocabulary: *nausea*, *colic*, *vomiting* and *diarrhea*. More expert terms such as *irritable bowel syndrome*, *irritable bowel,* or *dyspepsia* were used less frequently.

Messages were retrieved from 14 different generalized and specialized web forums. Extracted data mostly came from *Doctissimo* (182,647/198,866, 91.84% of messages; 27,415/36,989, 74.12% of users). The most frequently used data sources were *Aufeminin* (2325/36,989, 6.29% of web users), *Sante-medecine* (1375/36,989, 3.72% of web users), *Atoute.org* (1350/36,989, 3.65% of web users) and *Onmeda* (1341/36,989, 3.63% of web users).

**Table 1:** Most frequently used extraction keywords.

Keyword extraction (top 20)	English translation	Number of messages, n (%)
Mal au ventre	Abdominal pain	9011 (4.53)
Nausée	Nausea	2571 (1.29)
Colique	Colic	1795 (0.90)
Vomissement	Vomiting	1722 (0.87)
Gargouillis	Borborygmi	1698 (0.85)
Diarrhée	Diarrhea	1642 (0.83)
Ballonnement	Bloating	1548 (0.78)
Constipation	Constipation	1352 (0.68)
Des gaz	Gas	1070 (0.54)
Rot	Burp	996 (0.50)
Pet	Fart	847 (0.43)
Colopathe	IBS^a^	837 (0.42)
Colopathie fonctionnelle	IBS	757 (0.38)
Côlon irritable	Irritable bowel	609 (0.31)
Problèmes intestinaux	Bowel problems	599 (0.30)
Reflux gastrique	Acid reflux	488 (0.25)
Selles molles	Loose stools	452 (0.23)
Chiasse	Runs	402 (0.20)
Flatulence	Flatulence	364 (0.18)
Dyspepsie	Dyspepsia	338 (0.17)

^a^IBS: irritable bowel syndrome.

### Discussion Themes and Topics

A total of 18 topics of interest were identified on the basis of manual labeling and review of the data (Table 2). Although the most discussed topic was related to everyday life, the second most discussed topic was related to diet.

Topics were gathered into 6 main groups of clusters on their correlations: *consultations, diet, symptoms, quality of life, treatments* and *stress and symptoms*. The *symptoms* category was further subdivided into 3 subcategories: *abdominal pain, GERD,* and *digestion*. The 8 derived categories were used for user segmentation.

A second topic model was applied to the messages from the group of topics *diet*. This focus allowed the identification of a constellation of subtopics related to symptoms associated with diet (*nausea and vomiting, bloating and gastric reflux*), to the importance of adapting diet to avoid troubles (*diet as a solution to gastric troubles, recipes, balance in diet and efficiency of modifying the diet*), and to food intolerance *and intestinal microbiota (gluten, dairy products and intestinal flora*). This subtopic was rising in 2017 (ie, the relative number and number of posts discussing this topic), which is the last complete year of the analysis corpus.

Another topic model was applied to messages from the group of topics *stress and symptoms*. The main identified subtopic was addressing solutions to stress (*sport, courage* and *anxiety*). Other identified subtopics revealed a complex relationship between stress and symptoms of GI discomfort, as some subtopics were presenting GI symptoms as a cause of stress (*impact on social life and persistent gastric symptoms*) and stress as a cause of GI symptoms (*because of stress and pain because of problems*), sometimes during specific periods (*GI symptoms flare during exams or depending on the menstrual cycle*).

**Table 2:** List of modeled and merged topics.

Topics	Number of messages, n (%)	Number of users, n (%)	Group
Prediagnosis medical consultations	4752 (2.39)	2095 (5.66)	Medical consultations
Examinations for diagnostic purposes	3183 (1.60)	1283 (3.47)	Medical consultations
Postdiagnosis medical consultations	2795 (1.41)	1422 (3.84)	Medical consultations
Medical examinations	6852 (3.45)	4013 (10.85)	Medical consultations
Diet	12,802 (6.44)	4727 (12.78)	Diet
Food and IBS^a^	2211 (1.11)	988 (2.67)	Diet
Abdominal pain and nausea	12,385 (6.23)	6939 (18.76)	Symptoms—abdominal pain
Abdominal pain	8370 (4.21)	4130 (11.17)	Symptoms—abdominal pain
Gastroesophageal reflux disease	2001 (1.01)	672 (1.82)	Symptoms—GERD^b^
Gastroesophageal reflux disease and heartburn	6202 (3.12)	2337 (6.32)	Symptoms—GERD
IBS^a^	2538 (1.28)	1061 (2.87)	Symptoms—Digestion
Digestion	12,521 (6.30)	5290 (14.30)	Symptoms—Digestion
Digestive disorders in children	4520 (2.27)	2216 (5.99)	Symptoms—Digestion
Impact on everyday life	8628 (4.34)	3672 (9.93)	Quality of life
Everyday life	16,176 (8.13)	5902 (15.96)	Quality of life
Medication efficiency	6240 (3.14)	2825 (7.64)	Treatments
Information about the treatments	1561 (0.78)	856 (2.31)	Treatments
Stress and symptoms	5679 (2.86)	2971 (8.03)	Stress and symptoms

^a^IBS: irritable bowel syndrome.

^b^GERD: gastroesophageal reflux disease.

### User Segmentation

The algorithms based on regular expressions identified the gender for 14,441 users and the age for 4802 users. These results are presented in Table 3. The sex ratio was 0.20.

**Table 3:** Users’ characteristics: number of web users and relative number of web users (among 36,989 users).

Age range (years)	Women, n (%)	Men, n (%)	Unknown, n (%)
0-20	569 (1.54)	117 (0.32)	715 (1.93)
21-30	997 (2.70)	195 (0.53)	691 (1.87)
31-40	466 (1.26)	87 (0.24)	272 (0.74)
41-50	227 (0.61)	41 (0.11)	128 (0.35)
41-60	123 (0.33)	19 (0.05)	69 (0.19)
61 and over	42 (0.11)	13 (0.04)	31 (0.08)
Unknown	9647 (26.08)	1898 (5.13)	20,642 (55.81)

**Figure 4:**
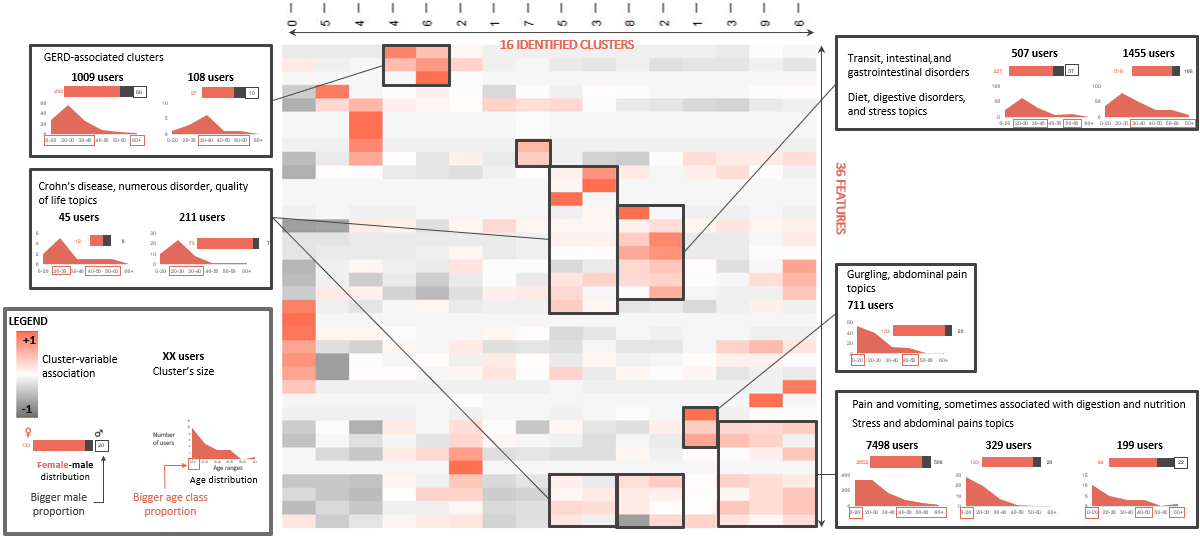
Heatmap presenting the results of the hierarchical clustering of web users on the basis of the discussed topics, symptoms, and activity on websites. GERD: gastroesophageal reflux disease.

A total of 12.98% (4802/36,989) of the users were characterized according to their age range (62,146/198,866, 31.25% of the messages), and 39.04% (14,441/36,989) of the users were characterized according to their gender (118,882/198,866, 59.78% of the messages). The nonsupervised, bottom-up, hierarchical clustering exhibited 16 different clusters according to the segmentation features presented in Multimedia Appendix 5. After clustering, the expression of these features enabled the visual identification of the expression of the features in a heatmap, which is presented in Figure 4. A review of this heatmap enabled the manual identification of clusters that are presented in Table 4. These clusters were labeled according to the expressed features, and only clusters of more than 100 web users were considered. Clusters are generally characterized by types of symptoms or diagnosed diseases (GERD, digestive disorders, stress and abdominal pain).

**Table 4:** List of main clusters of web users that were identified (36,989 users).^a^

Cluster name	Number of users, n (%)
Pains and vomiting, stress and abdominal pain	8026 (21.70)
Gastro intestinal disorders associated with diet, digestive disorders, and stress	1962 (5.30)
GERD^b^	1117 (3.02)
Borborygmi and abdominal pain	711 (1.92)
Crohn disease	256 (0.69)

^a^Clusters were named according to the features that were expressed in users’ messages.

^b^GERD: gastroesophageal reflux disease.

Two clusters were strongly associated with GI disorders. These 2 clusters were also associated with the groups of topics*, stress, symptoms,* and *diet*. Three clusters were strongly associated with pain, vomiting, and groups of topics*: stress and symptoms* and *symptoms—abdominal pain*. These clusters were associated with a younger population. More generally, clusters associated with undiagnosed symptoms were often associated with the *Stress* topic, whereas clusters associated with diagnosed diseases (GERD and Crohn disease) seemed to be associated with fewer stress features. The 4 clusters associated with the *Diet* topic were also associated with the group of topics *stress and symptoms*.

### Factors of GI Discomfort

The causality dictionary contained 170 terms. These causality terms were searched in the analysis corpus subset containing extraction keywords (29,935 messages). A total of 20,500 messages (corresponding to 10,848 users) were identified, and a new topic model was applied. The characteristic tokens and a sample of characteristic messages associated with each topic were manually reviewed to identify the themes addressed and the topics related to the causes of GI discomfort. In total, 10 topics of interest arose and were manually labeled and grouped according to the type of factors they expressed. This led to the identification of 7 different types of factors. The proportion of messages associated with these factors was calculated (Table 5). The factors that were mostly perceived by web users were related to the *psychological context* (psychological and social factors), followed by *diet* (nutritional factors and allergens or food intolerances) and *medical factors* (GI diseases, gynecological factors, and medical complications).

**Table 5:** Perceived factors of gastrointestinal discomfort. Proportions are calculated among the messages with causality terms (20,500 messages).

Factors and topics		Messages, n (%)
**Psychological factors**		4327 (21.11)
	Stress	2548 (12.43)
	Anxiety	2374 (11.58)
Nutritional factors		3224 (15.73)
Allergens		2857 (13.94)
**Diagnosed gastrointestinal diseases**		2697 (13.16)
	Digestive disorders	1516 (7.40)
	Medical examinations	1296 (6.32)
**Gynecological factors**		1898 (9.26)
	Obstetrical factors	1005 (4.90)
	Gynecological factors	980 (4.78)
Social factors		1568 (7.65)
Medical complications		1070 (5.22)

## Discussion

### Principal Findings

GI discomfort is actively discussed in French web forums, as shown in this study, which enabled the identification of 198,866 messages. In a subcorpus of web users who we were able to characterize according to age and gender, mostly women aged below 40 years were represented (Table 3). The gender and age distribution of most active users tended to mirror the higher prevalence of DGBIs such as IBS in younger women [47].

Users described how they were adapting their diet to avoid symptoms linked to perceived food intolerance (*gluten and dairy products*) associated with gut microbiota (*intestinal flora*). This is in line with the increasing number of reports in the literature about the controversial concept of nonceliac gluten sensitivity [48].

As stress was reported by users as both the cause and consequence of their GI symptoms, a focus on the *stress and symptoms* group revealed discussion topics related to the impact of stress on quality of life and solutions to reduce stress, such as physical activity. The role of psychological factors such as anxiety in eliciting or worsening GI symptoms is well established in the literature, both in the general population and in patients affected by DGBIs [49].

User segmentation led to the identification of 16 different classes, grouped into 6 main clusters. The classes associated with symptoms were generally associated with stress, with a stronger association in the case of abdominal pain. The 3 clusters associated with abdominal pain (attributed to diet or digestion) were associated with a younger population. In addition, in all groups of users reporting diet-related features, *Stress* and *Symptoms* topics were also expressed. This suggests a complex relationship between diet, stress, and symptoms of GI discomfort in a real-life setting. We believe that these results may appeal to researchers collecting dietary parameters in nutrition and clinical studies, as the monitoring of dietary intake and habits is important in prospective medical research studies [50]. Indeed, further context on meal intake (such as social and emotional context) should be collected to ensure that eating behavior and associated sentiments are accounted for. In a recent review, it was highlighted that emotion tracking is a lacking feature in most downloaded smartphone apps that are used for dietary assessment [51]. However, these tools could include features that may be used to examine emotions associated with meals in an observational setting at the population level. Such features would also be important to obtain further information on background diet, which is important when evaluating the efficacy of food and dietary interventions in research [52,53], especially in patients with DGBIs [54].

Our analysis identified 7 categories of factors of GI discomfort (psychological, nutritional, allergens, diagnosed GI diseases, gynecological, social, and medical complications), showing that food and psychological factors are perceived by web users as the major causes of GI discomfort. Identification of perceived factors revealed complex associations between food and health parameters. As an example, the use of *fibres* (fibers) keyword revealed a contrasted perception by web users, fibers being seen both as a solution to and cause of GI discomfort (associated with nutritional factors). As noted by another research team analyzing bowel disease–related tweets [55], web-based messages about foods and diet (in this case fiber, iron and magnesium) can be positively or negatively perceived depending on the conditions of web users. Regarding other keywords representing factors of food origin, milk, gluten, and fruits were some of the most frequently used terms.

### Comparison With Prior Work

The results of another study aimed at characterizing the inflammatory bowel disease community based on Twitter discussions during an 8-month period were published while this paper was being prepared [55]. This research team also identified that web users shared their experiences and looked for medical advice and that users’ discussions were mainly about inflammatory bowel disease symptoms, related diseases (including anxiety disorders), and foods and diets (including dietary interventions, such as gluten-free and probiotics). These findings are consistent with the main discussion topics that we identified in our corpus, even though the media source (tweets) and language (English) were different, and the studied indication (inflammatory bowel disease) was more specific for this study.

Recently, the smartphone app, *My Symptoms* [56], was completed by 163 participants to track food intake, psychological distress and GI symptoms in a research study aiming to identify the associations between these parameters. The results of this study were recently published by the research team [57], which described strong symptom-symptom associations, especially abdominal pain, bloating, gas-related discomfort, and psychological distress. All these parameters are topics or subtopics that were identified in our study; at the same time, we also noted an association between abdominal pain and stress.

We identified that topics related to medical consultations and medical examinations were frequently discussed, suggesting an important use of the health care system due to GI disorders, which is consistent with prior work [17]. When this paper was drafted, results of another study relying on another source of real-world information, the French National Health Data System (*Système National des Données de Santé*) [58], were published [59]. This study aimed to assess health care use in a specific case of IBS. This study also revealed an important use of the health care system by patients with IBS, also interestingly suggesting an important medical nomadism for these patients in France.

### Limitations

A limitation of this study is inherent to the particularities of web forums where web users do not necessarily reflect the characteristics of the general population. Although the important number of extracted messages could favor the variability of users’ characteristics, these results cannot be generalized to all patients affected by GI discomfort.

An extraction bias is associated with the considered data sources and keywords selected for analysis. Moreover, information found in messages cannot be interpreted as it would be from a questionnaire. Handling missing information is a key example: the fact that a piece of information is not found in messages does not mean that users did not experience it. For these reasons, it can be difficult to draw conclusions in cases of missing or unclear data. Another example is the identification of the age and gender of web users, which is not possible if not indicated in the source and not systematically identified in our study.

An additional limitation is the observational bias inherent to semantic analysis and natural language processing. The use of automatic analysis allows us to analyze a large amount of information but is subject to limitations arising from the abilities of the algorithms. Moreover, regarding topic models, the fact that topics must be manually labeled is also a source of bias.

The processing of lay language as source data prevents us from drawing further conclusions on the identified factors of GI discomfort that would require a high level of knowledge from web users. These factors are, therefore, presented as *perceived* factors in this paper as the assessment of their relationship with symptoms of GI discomfort results from self-assessment by web users. In addition, this analysis revealed several misconceptions, especially about factors of food origin. For example, web users may discuss food-allergic reactions but may refer to symptoms that are unlikely to be mediated by the immune system or compounds that are not known to be the cause of allergic reactions but rather of hypersensitivity or intolerance (eg, lactose). This is consistent with the results of a population-based survey published during this study, concluding that population-estimated prevalence of allergy was twice as important as the one estimated by physicians [60]. However, despite these limitations due to the analyses in lay language, our study confirmed a known and complex relationship between food, stress and psychological factors when considering online forums as a complementary source of real-world evidence.

### Conclusions

GI discomfort is an actively discussed topic in French web forums. When identified in a portion of active users, the gender and age of most active users tend to mirror the higher prevalence of DGBIs in women aged below 40 years. We were able to segment web users into several clusters corresponding to specific GI symptoms or diagnosed disorders and characterized by distinct demographic parameters and expression of variables related to stress. The main factors of GI discomfort as perceived by web users are food and psychological factors. This paper could benefit from a similar analysis based on additional sources to cover further languages (eg, English) to study the similarities and differences of the results at a larger scale and with different dietary and cultural backgrounds. To conclude, this innovative infodemiology approach has shown that identifying discussion topics associated with GI discomfort online is feasible and can serve as a complementary source of real-world evidence.

## Appendix

Multimedia Appendix 1 Corpus description.

Multimedia Appendix 2 Number of extracted messages and associated number of web users per data source.

Multimedia Appendix 3 List of keywords used for the extraction of messages.

Multimedia Appendix 4 List of features used for the segmentation of web users.

Multimedia Appendix 5 List of anatomical regions and pathophysiological mechanisms used for the segmentation of users.

Multimedia Appendix 6 List of causality terms used for the identification of perceived factors.

## Abbreviations

DGBI: disorders of gut-brain interaction
GERD: gastroesophageal reflux disease
GI: gastrointestinal
IBS: irritable bowel syndrome
LDA: latent Dirichlet allocation
MedDRA: Medical Dictionary for Regulatory Activities
TF-IDF: term frequency-inverse document frequency
