# Supplementary material for: Mapping and Modeling of Discussions Related to Gastrointestinal Discomfort in French-Speaking Online Forums: Results of a 15-Year Retrospective Infodemiology Study
Source: J Med Internet Res. 2020 Nov 3;22(11):e17247. doi: 10.2196/17247 (PMC7671840; doi:10.2196/17247)
Supplement: Multimedia Appendix 1 [file jmir_v22i11e17247_app1.docx]

1. Corpus description.

| Corpus name | Description of the corpus | Number of messages | Number of users |
| --- | --- | --- | --- |
| Analysis corpus | Messages related to GI discomfort | 198,866 | 36,989 |
| Messages associated with keywords | Messages containing extraction keywords corresponding to GI symptoms | 29,935 | 16,746 |
| Causality corpus | Messages containing GI symptoms AND causality terms | 20,500 | 10,848 |
