# Supplementary material for: Mapping and Modeling of Discussions Related to Gastrointestinal Discomfort in French-Speaking Online Forums: Results of a 15-Year Retrospective Infodemiology Study
Source: J Med Internet Res. 2020 Nov 3;22(11):e17247. doi: 10.2196/17247 (PMC7671840; doi:10.2196/17247)
Supplement: Multimedia Appendix 2 [file jmir_v22i11e17247_app2.docx]

1. Number of extracted messages and associated number of web users per data source.

| **Forums** | **Number of messages** | **Number of web users** |
| --- | --- | --- |
| Doctissimo | 182,647 | 27,415 |
| Aufeminin | 4,546 | 2,325 |
| Atoute | 2,579 | 1,350 |
| Sante Medecine | 1,761 | 1,375 |
| Onmeda | 1,695 | 1,341 |
| Futura-Sciences | 1,232 | 656 |
| Vulgaris Medical | 1,092 | 734 |
| Psychologies | 908 | 565 |
| Laxophobie | 823 | 219 |
| Forum Ados | 508 | 204 |
| Thyroide | 381 | 251 |
| Meamedica | 318 | 318 |
| Entrepatients | 188 | 113 |
| Psychoactif | 188 | 123 |
| **Total** | **198,866** | **36,989** |
