# Supplementary material for: Mapping and Modeling of Discussions Related to Gastrointestinal Discomfort in French-Speaking Online Forums: Results of a 15-Year Retrospective Infodemiology Study
Source: J Med Internet Res. 2020 Nov 3;22(11):e17247. doi: 10.2196/17247 (PMC7671840; doi:10.2196/17247)
Supplement: Multimedia Appendix 3 [file jmir_v22i11e17247_app3.docx]

1. List of keywords used for the extraction of messages

| **Keyword** | **Synonyms** |
| --- | --- |
| aérophagie |  |
| ballonnement | balonement / balloné |
| borborygme | borborisme |
| chiasse |  |
| chie mou |  |
| colique | collique |
| côlon irritable | colon irrité |
| colopathie |  |
| colopathie fonctionnelle |  |
| constipation |  |
| crampe au ventre |  |
| crampes abdominales | douleur de l'abdomen / douleur abdominale |
| dérangements intestinaux |  |
| des gaz |  |
| diarrhée |  |
| distension abdominale | ventre gonflé |
| dyspepsie | dispepsie, éructation, goût acide |
| flatulence |  |
| flatuosité |  |
| flatus |  |
| gargouille | gargouillis |
| gastroparesie |  |
| inconfort digestif |  |
| intestin irritable | intestin irrité |
| intestin paresseux |  |
| irritation du colon |  |
| la coulante |  |
| lourdeur dans le ventre |  |
| la courante |  |
| mal au ventre | maux de ventre / maux au ventre / mal de ventre |
| maladie colique | malade collique |
| maladie du colon |  |
| météorisme |  |
| nausée |  |
| paralysie gastrique |  |
| pas caca |  |
| pet |  |
| pression dans le ventre |  |
| problème de transit |  |
| problèmes gastrique |  |
| problèmes intestinaux | problèmes aux intestins |
| reflux gastrique |  |
| remontée acide |  |
| remontée gastrique | brulure d'estomac |
| rétention gastrique |  |
| rot |  |
| selles molles |  |
| stase gastrique |  |
| syndrome de l'intestin irritable | SII |
| syndrome du côlon irritable | SCI (suivi d'un espace) |
| transit paresseux |  |
| transit ralenti |  |
| trouble du transit |  |
| troubles fonctionnels coliques |  |
| troubles fonctionnels du colon |  |
| tympanisme |  |
| ventre grogne | ventre qui grogne, grondement d'estomac |
| vomissement | vomir |
