# Supplementary material for: Mapping and Modeling of Discussions Related to Gastrointestinal Discomfort in French-Speaking Online Forums: Results of a 15-Year Retrospective Infodemiology Study
Source: J Med Internet Res. 2020 Nov 3;22(11):e17247. doi: 10.2196/17247 (PMC7671840; doi:10.2196/17247)
Supplement: Multimedia Appendix 4 [file jmir_v22i11e17247_app4.docx]

1. List of features used for the segmentation of web users.

| **SYMPTOMS** | **TOPICS** | **ACTIVITY** |
| --- | --- | --- |
| Gastric disorders | Gastroesophageal reflux | doctissimo thread |
| Esophageal disorders | Quality of life | number of posts |
| Crohn | Food | number of discussions |
| Rectal disorders | Digestion | activity period |
| Intestinal disorders | Medical appointment | last post published |
| Gastrointestinal disorders | Treatment | mean order of author's posts in the discussions |
| Digestive transit disorders | Abdominal pains | publication period: fall |
| Deficiency | Stress | publication period: spring |
| Vomiting |  | publication period: summer |
| Intolerance |  | publication period: winter |
| Appetite disturbance |  | first post published |
| Bowel sounds |  | mean post sizes |
| Flatulence |  | mean order of author's posts in the discussions |
| Biological markers |  |  |
| Pains |  |  |
