# Supplementary material for: Mapping and Modeling of Discussions Related to Gastrointestinal Discomfort in French-Speaking Online Forums: Results of a 15-Year Retrospective Infodemiology Study
Source: J Med Internet Res. 2020 Nov 3;22(11):e17247. doi: 10.2196/17247 (PMC7671840; doi:10.2196/17247)
Supplement: Multimedia Appendix 5 [file jmir_v22i11e17247_app5.docx]

1. List of anatomical regions and pathophysiological mechanisms used for the segmentation of users.

| **Anatomical regions and pathophysiological mechanisms** |
| --- |
| Gastric disorders |
| Rectal/anus disorders |
| Esophageal disorders |
| Intestinal problems |
| Gastrointestinal disorders |
| Crohn |
| Intolerances |
| Deficiencies |
| Biological markers |
| Others |
| Flatulence |
| Transit disorders |
| Pains |
| Vomiting |
| Appetite disorder |
