# Supplementary material for: Mapping and Modeling of Discussions Related to Gastrointestinal Discomfort in French-Speaking Online Forums: Results of a 15-Year Retrospective Infodemiology Study
Source: J Med Internet Res. 2020 Nov 3;22(11):e17247. doi: 10.2196/17247 (PMC7671840; doi:10.2196/17247)
Supplement: Multimedia Appendix 6 [file jmir_v22i11e17247_app6.docx]

1. List of causality terms used for the identification of perceived factors.

| **Causality term** | **Position of the analysed segment of phrase** |
| --- | --- |
| a cause de | after |
| a cause d | after |
| a chaque fois que | after |
| a chaque fois qu | after |
| a l origine de | before |
| a l origine d | before |
| a la suite de | after |
| a la suite d | after |
| abouti | before |
| aboutit | before |
| abouti a | before |
| abouti aux | before |
| abouti au | before |
| cause des | before / after |
| cause du | before / after |
| cause de | before / after |
| cause d | before / after |
| causait des | before / after |
| causait du | before / after |
| causait de | before / after |
| causait d | before / after |
| causent des | before / after |
| causent du | before / after |
| causent de | before / after |
| causent d | before / after |
| causaient des | before / after |
| causaient du | before / after |
| causaient de | before / after |
| causaient d | before / after |
| compte tenu de | after |
| compte tenu du | after |
| compte tenu des | after |
| compte tenu d | after |
| des lors | after |
| des que | after |
| donne lieux | before |
| donne lieu | before |
| donnent lieu | before |
| donnent lieux | before |
| du fait de | after |
| du fait du | after |
| du fait d | after |
| du fait que | after |
| en lien | after |
| en raison | after |
| en rapport | after |
| engendre | before |
| engendrent | before |
| entraine de | before |
| entrainent de | before |
| entraine d | before |
| entrainent d | before |
| entraine du | before |
| entrainent du | before |
| entraine des | before |
| entrainent des | before |
| entrainait de | before |
| entrainaient de | before |
| entrainait d | before |
| entrainaient d | before |
| entrainait du | before |
| entrainaient du | before |
| entrainait des | before |
| entrainaient des | before |
| engendrait | before |
| engendraient | before |
| est associe | after |
| est associee | after |
| est du | after |
| est due | after |
| était associe | after |
| etait associee | after |
| étaient associes | after |
| etaient associees | after |
| était du | after |
| était due | after |
| etaient dus | after |
| etaient dues | after |
| etant donne | after |
| etant donne que | after |
| lie a | after |
| lie au | after |
| liee a | after |
| liee au | after |
| lies a | after |
| lies au | after |
| liees a | after |
| liees au | after |
| lie aux | after |
| liee aux | after |
| lies aux | after |
| liees aux | after |
| lorsque | after |
| me fait | before |
| m a fait | before |
| m engendre | before |
| m engendrent | before |
| m occasionne | before |
| m occasionnent | before |
| m occasionnait | before |
| m occasionnaient | before |
| me cause | before |
| me causent | before |
| me causait | before |
| me causaient | before |
| me cree | before |
| me creent | before |
| me creait | before |
| me creaient | before |
| me declenche | before |
| me declenchent | before |
| me declenchait | before |
| me declenchaient | before |
| me donne | before |
| me donnent | before |
| me donnait | before |
| me donnaient | before |
| me produit | before |
| me produisent | before |
| me produisait | before |
| me produisaient | before |
| me provoque | before |
| me provoquent | before |
| me provoquait | before |
| me provoquaient | before |
| me rend | before |
| me rendent | before |
| me rendait | before |
| me rendaient | before |
| occasionne | before |
| occasionnent | before |
| occasionnait | before |
| occasionnaient | before |
| par suite de | after |
| par suite d | after |
| parce que | after |
| parce qu | after |
| provoque | before |
| provoquent | before |
| provoquait | before |
| provoquaient | before |
| quand je | after |
| quand j | after |
| sont associes | after |
| sont associees | after |
| sont dus | after |
| sont dues | after |
| sous l effet de | after |
| sous l effet d | after |
| sous l effet du | after |
| sous l effet des | after |
| suite a | after |
| suite au | after |
| suite aux | after |
| vient de | after |
| vient du | after |
| vient des | after |
| vient d | after |
| venait de | after |
| venait du | after |
| venait des | after |
| venait d | after |
| viennent de | after |
| viennent du | after |
| viennent des | after |
| viennent d | after |
| venaient de | after |
| venaient du | after |
| venaient des | after |
| venaient d | after |
